# A Practical Treatise on Diseases of the Skin

**Published:** 1888-07

**Authors:** 


A Practical Treatise on Diseases of the Skin. By John V. Shoemaker, A. M., M. D. New York: D. Appleton & Co.

To the average practitioner of medicine and surgery, the domain of skin diseases is a vast and unexplored wilderness. To three-fourths of this region, the general name of eczema is applied; to the other one-fourth, psoriasis.

With such a simple and primitive nosology, it is no wonder that the therapeutic measures so often fail.

Dr. Shoemaker has sought to lead us to a knowledge of diagnosis and rational treatment. Writing from the standpoint of the general practitioner, he has purposely avoided a discussion of mooted points in etiology and pathology, and given us, in rather a dogmatic way, a concise and clear view of his subject. The book possesses a special value, because it embodies the author's own experience in the therapeutics of skin diseases. The illustrations are abundant, and in the main very good. Some of the colored plates are open to criticism, possessing, as they do, a vividness of color rarely encountered in life. This, however, is a minor defect; otherwise the work is excellent, and we take pleasure in commending it to the profession.

